# Daidzein improves neuronal health and alleviates inflammation and apoptosis through BDNF and estrogen receptors in the hippocampus of ovariectomized rats

**DOI:** 10.22038/ijbms.2025.82074.17758

**Published:** 2025

**Authors:** Asma Neisy, Zahra Khoshdel, Farhad Koohpeyma, Atefeh Seghatoleslam, Zohreh Mostafavi-Pour, Sanaz Alaee, Fatemeh Keshavarzi, Saeed Shokri, Fatemeh Zal

**Affiliations:** 1 Department of Biochemistry, School of Medicine, Shiraz University of Medical Sciences, Shiraz, Iran; 2 Research committee, endocrine and metabolism research center, Shiraz University of Medical Sciences, Shiraz, Iran; 3 Reproductive Biology Department, School of Advanced Medical Sciences and Technologies, Shiraz University of Medical Sciences, Shiraz, Iran; 4 Autophagy Research Center, Department of Biochemistry, School of Medicine, Shiraz University of Medical Sciences, Shiraz, Iran; 5 School of Medical Sciences, Faculty of Medicine and Health, University of Sydney, Sydney, New South Wales, Australia; 6 Infertility Research Centre, Shiraz University of Medical Sciences, Shiraz, Iran

**Keywords:** CA1 region, Dentate Gyrus, Estrogen deficiency Hippocampal, Menopause, Ovariectomy

## Abstract

**Objective(s):**

Isoflavone Daidzein (DDZ) has emerged as a promising alternative to hormone replacement therapy (HRT) for ameliorating estrogen deficiency (ED). However, the stereological and molecular mechanism of its effects in the OVX-hippocampus are unclear. We studied the impact of DDZ on stereological changes, estrogen receptor (ERs) expression, BDNF, GSK-3β, and inflammatory and apoptosis-related genes in the hippocampus of ovariectomized rats, compared to 17β-estradiol (E2).

**Materials and Methods:**

OVX rats were treated with DDZ or E2. The stereological analysis assessed the total volume and number of pyramidal and granular neurons in the hippocampus CA1 and DG subregions. Expression of proinflammatory cytokines, apoptotic-related genes, ERs, and BDNF genes was evaluated using Real-Time PCR, and the GSK-3β phosphorylation level was measured by western blot analysis.

**Results:**

DDZ has effectively increased the volume and total number of pyramidal neurons in the CA1 region, the expression of ERα, ERβ, BDNF, and Bcl-2 genes, and the phosphorylation rate of GSK-3β protein. However, the effect of DDZ on the DG region, ERα, and BDNF genes was not significant in comparison with E2; DDZ significantly suppressed the expression of TNF-α, IL-6, and the Bax/Bcl2 ratio compared with OVX rats.

**Conclusion:**

DDZ effectively reversed the stereological changes in the CA1 region by stimulating BDNF gene expression, increasing the phosphorylation ratio of the GSK-3β protein, and modulating inflammatory and apoptotic pathways. Although its effects on the DG region, BDNF, and ERα molecules were less significant than E2, DDZ could still be a promising candidate for ameliorating ED.

## Introduction

17-β estradiol (E2) is a powerful regulator of brain hemostasis and neuronal health. Through binding to its specific nuclear receptors, Estrogen Receptor α (ERα) and Estrogen Receptor β (ERβ), which are differently distributed in various brain regions, E2 initiates a cascade of molecular events that influence vital processes such as synapse formation, cell signaling pathways, neurotrophin systems, and neurogenesis (1, 2). It is now well established that the hippocampus is an early target structure for these effects (1, 3). The human hippocampus is fundamental in creating memories, as well as cognition formation (4, 5), which is carried out by a group of pyramidal cells in the CA1, CA2, and CA3 regions, along with Dentate Gyrus (DG) granule cells (2). Previous research has confirmed that E2 potentially enhances neuronal cell proliferation (6) and synapse formation in all the mentioned regions, especially in hippocampal-CA1 and DG regions (7, 8). 

It has been suggested that the neuroprotective effects of E2 can occur through the regulation of a variety of molecules that play a central role in the hippocampus’s cognitive function, mood stability, and synapse plasticity, including Brain-Derived Neurotrophic Factor (BDNF)(9) and Glycogen synthase kinase 3β (GSK-3β). The BDNF molecule is a member of the neurotrophic factors family, which is widely distributed throughout the brain in diverse human cell types and promotes axon growth and the survival of various neuron clusters (10). The presence of ERE sequences on the BDNF gene suggests that BDNF is part of the estrogenic effects of transduction (9, 11). Additional research has revealed that E2 can also impact the survival of the neurons by adjusting the activity of the GSK-3β molecule, which is present at high levels in the central nervous system (12). Following menopause, the phosphorylated form of GSK3β significantly decreases, leading to the activation of oxidative stress, neuroinflammation, and apoptosis (13). E_2_ has also been found to control neuronal inflammation and apoptosis by regulating the expression of Tumor Necrosis Factor α (TNF-α), Bcl-2, and Bax gene expression in postmenopausal rats (14). Hence, a decline in estrogen levels during reproductive age or menopause has been associated with impairment of learning, memory, and cognition ability (15, 16) and the incidence of neurodegenerative diseases (NDD) controlled by HRT. 

One of the clinical strategies to minimize pathological alterations associated with ED is to use HRT. HRT in postmenopausal women or those who have undergone adnexectomy can help prevent osteoporosis. It can also alleviate the specific symptoms of menopause, including vasomotor symptoms, sexual dysfunction, hot flashes, and urogenital atrophy; however, its effectiveness is severely limited by its detrimental effects, such as the higher risk of breast cancer, cerebrovascular disease (17), and endometrial carcinoma (18), justifying the need for therapeutic alternatives. Thus, nonsteroidal estrogen-like compounds have garnered significant attention as safe and effective therapeutic alternatives. 

Phytoestrogens are a popular alternative to HRT and the most potent natural bioactive compounds with proven scientific benefits.; They were found effective in preventing menopausal symptoms such as osteoporosis (19), cardiovascular disease, and insulin resistance (20). Phytoestrogens are usually absorbed in the body through dietary soy and its derivative compounds. Physiologically safe and achievable doses of phytoestrogen have the potential to emulate the specific neuroprotective effects of 17β-estradiol. It has been reported that daidzein, primarily present in soy and many unfermented foods (21), can activate ERs (22) and protect the primary hippocampal neurons from oxidative stresses induced by glutamate or β-amyloid (23). Wei *et al*. found that treatment with daidzein improved the memory and learning impairments caused by ICV-STZ. Additionally, it restored the levels of malondialdehyde, catalase, and superoxide dismutase and reduced glutathione to their normal values (24). Considering the many effects of estrogen on the brain, an alternative therapy for HRT should be able to carry out most of these activities while having fewer side effects compared to HRT. Despite what has been mentioned, there is still limited knowledge about the potential ability of daidzein to regulate the hippocampal vital molecules and stereological changes caused by estrogen deficiency in the hippocampus of ovariectomized rats compared to E2. Therefore, we aimed to study the effect of treatment with 20 mg/kg/day DDZ on the expression of ERα, ERβ, BDNF, TNF-α, IL-6 Bcl-2, and Bax genes, GSK3-β protein level, and hippocampal CA1 and DG subregions stereological changes in ovariectomized rats in comparison with 10 μg/kg E2.

## Materials and Methods

### Drugs and chemicals

Daidzein (FD10005) was purchased from BIOSYNTH (Staad, Switzerland) and 17-β-estradiol was purchased from Abureyhan Pharmaceutical Company (Tehran, Iran). BIOZOL Total RNA Extraction reagent (BSC51M1) for RNA extraction was purchased from Zhejiang, China. Revert Aid First Strand cDNA Synthesis Kit were from Pars Tous biotechnology, primary antibodies, GSK3β and p-GSK-3β (Ser 9), and anti-rabbit horseradish peroxidase (HRP) conjugated secondary antibody were purchased from Cell Signaling (Massachusetts, USA) and ECL western blotting substrate kit was purchased from Abcam, USA. E2 ELISA kit assay was purchased from Ideal Tashkhis Atieh Estradiol (Iran, Cat No: 2824-96). 

### Animals

The experimental protocol adhered to the ARRIVE guidelines and was approved by the ethical committee of Shiraz University of Medical Sciences for Animal Welfare and Studies (Ethic code: IR.SUMS.AEC.1400.021).

This study was conducted on 50 female Sprague Dawley rats weighing 200–250 g each, obtained from the Animal Laboratory Center at Shiraz University of Medical Sciences, Shiraz, IRAN. The rats were treated with the utmost care in a constant environment with a temperature of 23 ^°^C and humidity of 55% and had access to food and water *ad libitum*. Two weeks before the experiment, rats were randomly assigned to five groups (n=10) and underwent surgery. Two groups had surgery without removing ovaries, and they received just vehicle (90% corn oil and 10% ethanol)(25). They were defined as the Sham+Vehicle (Sham for short) and DDZ+Vehicle (DDZ for short) groups. Three other groups that had surgery and bilateral ovariectomy: The OVX+Vehicle (OVX for short), The OVX+DDZ group received 20 mg/kg/day DDZ (26). The OVX+E2 group (Positive control) was treated with 10 µg /kg/day of E2 (27, 28) S.C. injection. The dose, duration of DDZ and E2 administrations, and the vehicle were selected based on previous studies. All treatments were started two weeks after ovariectomy and a week after checking vaginal smear. After 50 consecutive days of treatment (29), all animals were humanely killed, and their hippocampus was collected and kept under appropriate storage conditions at -80 ^°^C.

### Ovariectomy procedure

Bilateral ovariectomy was performed using the dorsal method under anesthesia with ketamine (60 mg/kg) and xylazine (5 mg/kg). First, the dorsal area of the animals was shaved and cleaned with 70% ethanol. A single 2 cm incision was made under sterile conditions on the lower abdominal region between the umbilicus. The abdomen’s muscles and skin were opened, and both ovaries were removed. After extracting the ovaries, 1-2 ml of physiological saline solution was poured into the abdomen and muscles. The incision was sutured closed, and lidocaine and tetracycline ointments were applied locally to the incision site (30).

### Sample collection

The weights of rats were measured a day after ovariectomy and at the end of the study (data not given). After 50 consecutive days of treatment, blood samples were collected from the heart, and the obtained serum was kept under appropriate storage conditions for E2 analysis; then, the rats were killed. Half of the hippocampus tissue from each rat was removed and stored at -80 ^°^C for RNA extraction, real-time polymerase chain reaction (RT‐PCR), and western blot analysis. Another was kept in 10% formaldehyde for histological and immunofluorescent assay.

### Serum E2 assay

Blood samples were centrifuged at 3000g for 15 min at 4 ^°^C, and the supernatants were used as serum samples. Serum E2 level was determined using a commercially available enzyme-linked immunosorbent assay (Ideal Tashkhis Atieh Estradiol) kit according to the manufacturer’s protocol.

### RNA extracting method, cDNA synthesis protocol, and Real-Time PCR

The total RNA from the hippocampus tissue was extracted using a BIOZOL Total RNA Extraction reagent under the manufacturer’s protocol (BSC51M1, Zhejiang, China). The integrity of the extracted RNA molecules was verified by running the purified RNA on a 1.5% agarose gel stained with GelRed. RNA was then utilized to create cDNA via the Revert Aid First Strand cDNA Synthesis Kit (Pars Tous biotechnology). Quantitative analysis of the genes of interest was conducted using Real-Time PCR with GAPDH as the reference gene. The ‐∆∆ 2 Ct method was used to calculate relative gene expression levels. SYBR Green-based Real-Time PCR was done using The Quant Studio Real-Time PCR system (31). Table 1 presents the primer sequences employed in this study.

### Protein extraction and Western blotting

Protein isolation was performed using 20 mg of protein extracted from the rat’s hippocampus. The RIPA solution buffer (NaCl (150mM), Nonidet P-40 (1%), Sodium deoxycholate (DOC)(0.5%), Sodium dodecyl Sulfate-polyacrylamide (SDS)(0.1%), Tris (50 mM) with pH 7.4, 2.5 ml protease inhibitor cocktail, and 3 ml phosphatase inhibitor cocktail per 20 mg tissue was used for extraction. After the extraction procedure, equal amounts of protein were mixed with loading dye, boiled for 5 min, and separated with sodium dodecyl sulfate-polyacrylamide gel electrophoresis (SDS-PAGE) 15%. The protein was transferred onto a nitrocellulose membrane and blocked bovine serum albumin (BSA) 5% at room temperature for two hours. The membrane was then exposed to primary antibodies, GSK3β (Cell Signaling, 9315) and p-GSK-3β (Ser 9)(Cell Signaling, 9322), at a 1:1000 dilution in TBS containing 1% BSA and Tween-20. After the blots were rinsed three times in TBS-T to visualize the protein bands, an anti-rabbit horseradish peroxidase (HRP) conjugated secondary antibody (Cell Signaling Technology, USA) was used with 1:2000 dilution to incubate the membranes for an hour. The ECL western Blotting Substrate Kit from Abcam, USA, and the ChemiDoc™ MP Imaging System from Bio-Rad, USA, were utilized to capture the photos of the blots. Image Analysis Software (Bio-Rad, USA) was used to determine each band, and then the level of the target protein was calculated as the ratio of each target band/tubulin. The average ratio for each band/Tubulin was obtained from two membranes (32).

### Stereological analysis

*Volumetric analysis*

The brain volumetric estimation procedure was performed by preparing about 15-20 sections from each hippocampus. The sections were selected in a systematic random manner, and each selected section was 26 μm thick. The selected sections were stained with a modified Giemsa stain, and CA1 and DG of the hippocampus were photographed on a PC screen using Olympus BH-2 (Japan light microscopy) with a digital color camera attachment (Sanyo VVC-6975P, Japan). The unbiased Cavalieri method and the light microscopic images were used to estimate the hippocampus volume. A point-counting test grid was used to approximate the areas sectioned in the hippocampus. The point density of the grid was designed to achieve an appropriate coefficient of error (CE) for places of interest in the images of the serial sections. The coefficient of variation (CV) was estimated according to Gundersen and Jensen’s formula (33). The test grid consisted of a systematic array of points randomly placed on the PC screen. The volume of each hippocampus was estimated using the following formula:

The sum of the area of the chosen structure “ΣA (sections)” (CA1 and DG) was multiplied by the distance between the sampled sections, considered as “d.

V (CA1, dentate gyrus) = ΣA(sections) × d

*Estimation of the CA1 pyramidal cells and the dentate gyrus granular cells*

**Figure 1:**
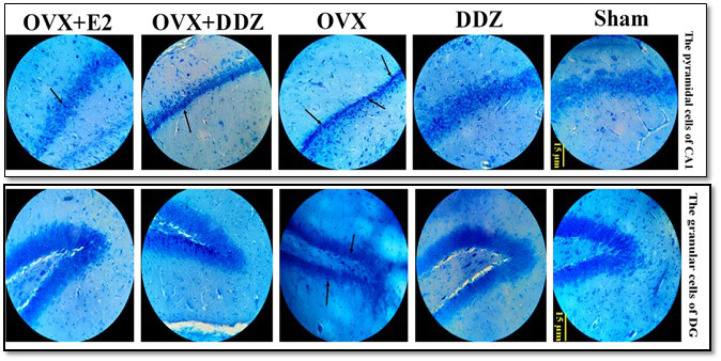
Effect of daidzein on the ovariectomy-induced stereological changes in the rat hippocampus

The total number of granular cells in DG and CA1 pyramidal cells have been evaluated by using a micro-actuator and video microscopy system, following the “optical dissector method” (shown in Figure 1). The von Bartheld method was used to plot the Z-axis distribution of neuron nuclei, counting the total number of neurons by multiplying the numerical density of “Nv” and V (CA1, DG) to determine t-guard zones (34): 

 The “ΣQ” is the total number of nuclei, “ΣP” is the total number of points that hit the assessed tissue, “af” is the frame area, “h” is the height of the Dissector, “t” is the mean section thickness, and “BA” is the microtome setting (35).

### Statistical analysis

SPSS software version 16 (IBM Corporation, Armonk, NY, USA) and GraphPad software version 6 (San Diego, CA, USA) were used for statistical analysis. All data were statistically reported as mean±SEM. The data normality was checked with the Kolmogorov-Smirnov test. A three-way ANOVA statistical analysis with Tukey’s *post hoc* test was applied for normally distributed data. The Kruskal-Wallis’s test, followed by the Mann-Whitney U test, was used for non-normally distributed data and the data of protein expression densitometric. *P*<0.05 indicates significant differences between groups. 

## Results

### Effects of ovariectomy and treatments on serum 17β-estradiol levels

Serum levels of 17β-estradiol were significantly reduced in OVX rats compared to the sham group (*P*<0.05). No significant change was observed in the 17β-estradiol levels of the ovariectomized rats before treatments (Table 2).

### Real-time PCR results

RT-PCR was used to evaluate the hippocampus expression of BDNF, TNF-α, IL-6, Bax, Bcl-2, ERβ, and ERα genes. 

*Daidzein enhanced the expression of hippocampal estrogen receptor genes*

**Figure 2:**
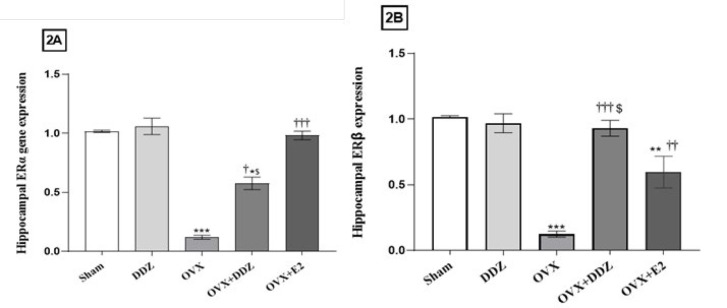
Effect of daidzein on the expression of ERα and Erβ genes in the hippocampus

The expression of ERα and ERβ mRNA in the hippocampal tissue was assessed by Real-Time PCR and compared between all groups. As shown in Figure2.A and Figure2.B, the ovariectomy-induced ED in the OVX rats significantly suppressed the expression of ERα and Erβ genes compared to the sham group (*P*<0.001). Our results showed that 20 mg/kg/day of DDZ could remarkably increase the expression of ERα and ERβ by 5 times and 9 times, respectively, compared with OVX rats (*P*<0.01). The most surprising aspect of the data is that the effect of DDZ on the Erβ gene expression was significantly more than that of E2 in the mentioned gene (*P*<0.05). Further analysis showed that E2 (OVX+E2) was more efficient in the induction of the ERα gene compared to daidzein (OVX+DDZ) (*P*<0.05).

*Daidzein induced the expression of the hippocampal BDNF gene*

**Figure 3:**
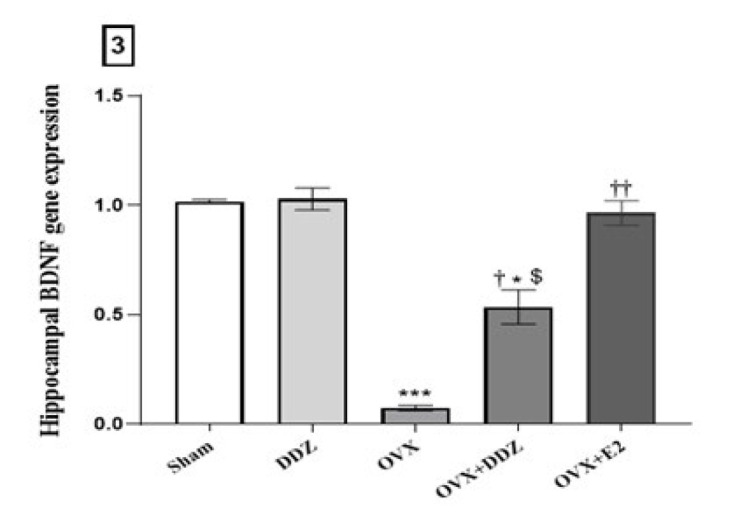
Effect of daidzein on the hippocampal BDNF gene expression

Figure 3 presents the result obtained from the preliminary analysis of hippocampal BDNF mRNA expression in five experimental groups. There is a clear trend of decreasing the expression levels of the BDNF gene in the ovariectomized rats compared to the sham group (*P*<0.001). Further analysis showed that consumption of DDZ significantly reversed this change in the OVX+DDZ rats compared with OVX (*P*<0.01). The BDNF gene expression level in OVX+DDZ rats was 7.14 times higher than in OVX conditions. A Comparison between the effect of E2 and DDZ on BDNF gene expression showed statistically significant differences between the effects of these two treatments (*P*<0.05). The Real-Time PCR data revealed that E2 had enhanced BDNF gene expression 14 times compared to the OVX rats and was more potent than DDZ (*P*<0.01). 

*Daidzein suppressed the expression of the inflammatory cytokines*

**Figure 4:**
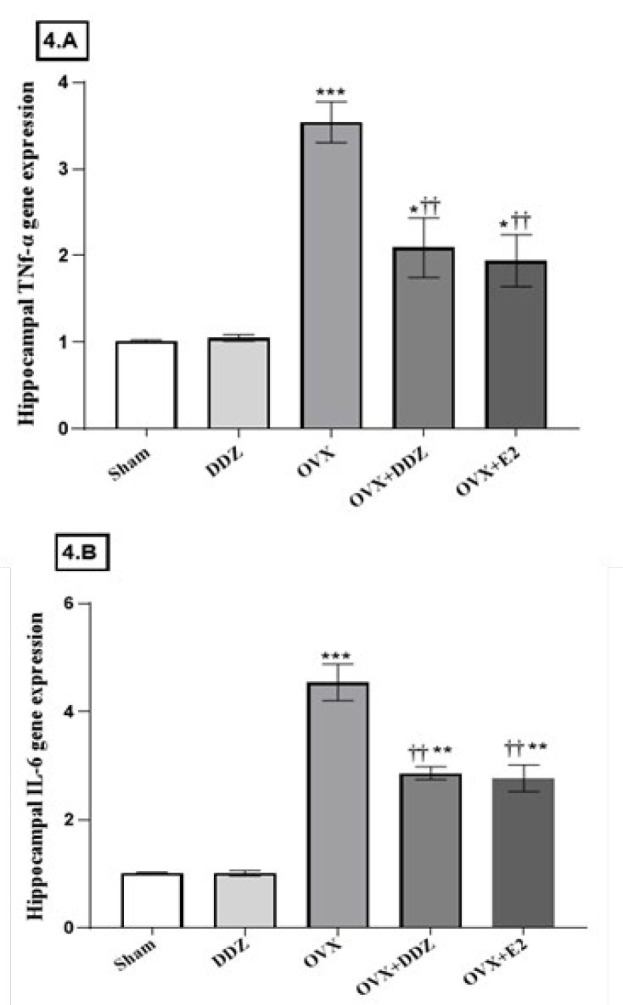
Effect of daidzein on the expression of inflammatory cytokines

The results of one-way ANOVA revealed that the expression level of the hippocampal TNF-α and IL-6 genes dramatically increased in the OVX rats compared to the sham group, respectively (*P*<0.001). As shown in Figure 4.A treatment with DDZ significantly suppressed the expression of the TNF-α mRNA levels compared to the OVX rats (*P*<0.01). Also, DDZ significantly decreased the expression levels of hippocampal IL-6 OVX (*P*<0.01)(Figure 4.B). There were no statistically significant differences between the effects of E2 and DDZ in this regard (Figures 4 A and B).

*Daidzein reversed the expression of the apoptosis-related genes and apoptotic ratio.*

**Figure 5:**
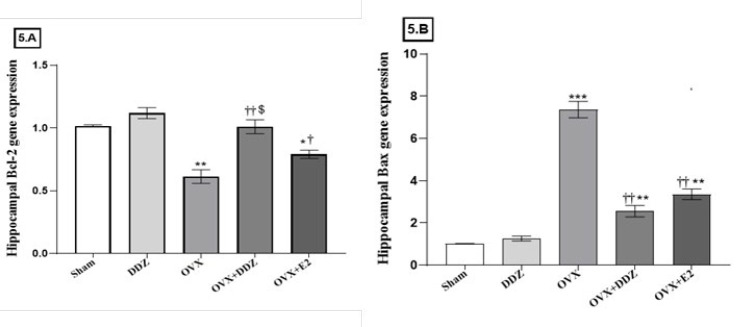
Effect of daidzein on the Bcl-2 and Bax genes expression

Figures 5. A and B depict the expression rate of Bcl-2 and Bax genes in the hippocampus of all examined groups. Our results showed that the expression level of the Bcl-2 gene significantly decreased in the OVX group compared to the sham group (*P*<0.01). Further analysis revealed a statistically significant elevation in the Bcl-2 gene expression in the OVX+DDZ group to such an extent that there were no statistically significant differences between the OVX+DDZ group and the sham rats. These changes were statistically significant when compared to the OVX rats (*P*<0.01)(Figure 5.A). As illustrated in Figure 5.B, the expression level of Bax, as an apoptotic marker, was 7.3 times higher in the OVX rats compared to the sham rats (*P*<0.01). Consumption of the daidzein in the OVX+DDZ rats suppressed this gene compared to the OVX rats (*P*<0.01). There were also significant differences in the effects of DDZ and E2 in inducing the Bcl-2 gene (*P*<0.05).

The hippocampus Bax/Bcl-2 mRNA ratio was calculated and shown in Table 3. The ovariectomy caused a sharp increase in the apoptotic ratio compared with the sham rats (*P*<0.01). This ratio significantly decreased in the OVX+DDZ hippocampus and reached about 1/6 of the amount in the OVX group (*P*<0.05). A statistically significant difference in the Bax/Bcl2 ratio between the OVX+DDZ and OVX+E2 rats also were seen (*P*<0.05).

### Western blot analysis results

*Daidzein enhanced the level of pGSK-3β protein in the hippocampus*

**Figure 6:**
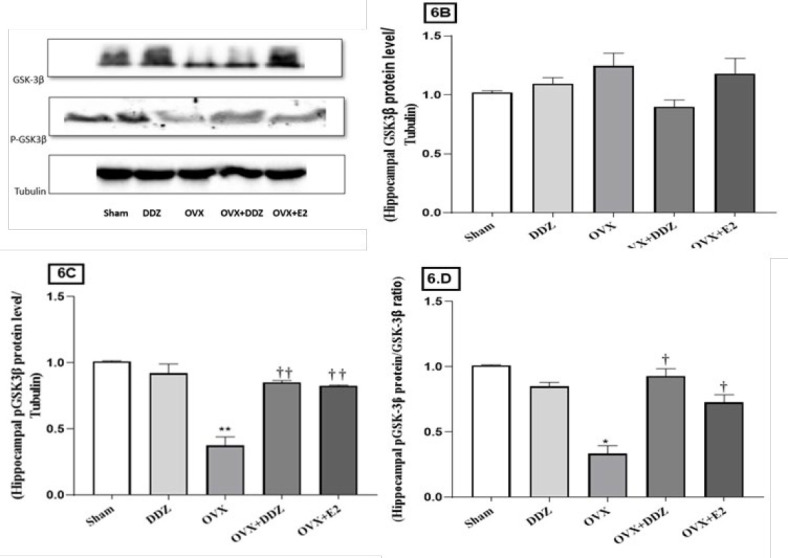
Effect of daidzein on the hippocampal GSK-3β protein phosphorylation rate

The results of the Western blot analysis technique are shown in Figures 6. A, B, C, and D. Our research aimed to evaluate the effect of ED on the total level of hippocampus GSK-3β protein. Despite the elevated level of GSK-3β in the ovariectomized group, no statistically significant effect on the mentioned protein level was found in the hippocampus of the OVX rats compared with the sham rats (*P*>0.05). In this study, we found that administration of DDZ reduces the GSK-3β protein level in OVX+DDZ rats; however, this effect was not statistically significant when compared to the OVX or OVX+E2 groups (Figure 6.B)(*P*>0.05). Further investigation showed a statistically significant difference between the GSK-3β protein phosphorylation (pGSK3-β) level in the OVX group compared to the sham group *(P*<0.01*)*. Treatment with DDZ enhanced the phosphorylation rate of GSK-3β protein compared with the ovariectomy condition, although its effect was not significantly different from the effect of E2 *(*Figure 6 D). In the following, it was found that the ratio of GSK-3β phosphorylation to non-phosphorylation protein in the OVX group dramatically decreased compared to control rats (*P*<0.05)(Figure 6.D) in both treated groups receiving DDZ and E2 this ratio was significantly elevated compared to the OVX rats, by 3 times and 2 times, respectively *(P*<0.05). 

### Stereological analysis results

*Daidzein ameliorated the stereological changes of hippocampal CA1 and DG regions*

**Figure 7:**
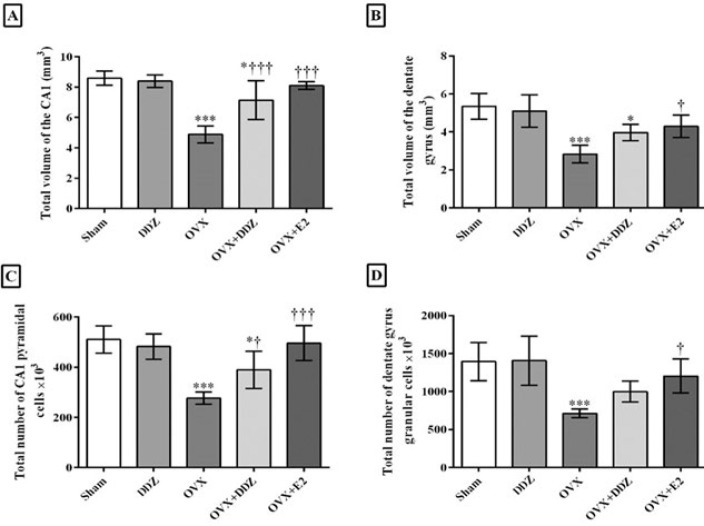
Effects of DDZ on the ovariectomy-induced stereological changes in hippocampal CA1 and DG subfields

Figure 7 illustrates a summary statistic for hippocampus stereological changes after ovariectomy and treatment with DDZ. The analytic data showed that ovariectomy caused a significant decline in the total volume of the DG and CA1 subregions. The total number of pyramidal and granular neurons was significantly decreased in the CA1 and DG regions of the OVX hippocampus, respectively, compared to the sham group (*P*<0.001)(Figure 7.A and Figure 7.B). Further analysis revealed that treatment with 20 mg/kg/day DDZ caused a 2.6-fold increase in the CA1 volume compared to that of the OVX rats (*P*<0.01) (Figure 7.A). DDZ also induced the proliferation of CA1 pyramidal neurons; the total number of pyramidal cells in the OVX+DDZ group increased 2.8-fold compared with the OVX rats (*P*<0.05) (Figure 7.C).

 As shown in Figures 7.B and 7.D, no statistically significant differences between the volume of the DG region and the total number of granular neurons in this area were found in the DDZ + OVX compared to OVX rats. Additionally, no significant differences were found between the effect of E2 and DDZ in hippocampal subregions volume and total number of the mentioned neurons.

## Discussion

Estrogen deficiency is defined as a chronic decline in endogenous estrogen levels, which has severe consequences for affected women. The most important changes involve cognitive functions. This sudden decline in E2 levels was suggested to be involved in female age-related neurodegenerative changes. Our real-time PCR data showed that it can lead to reduced expression of both estrogen receptor genes. Reducing the expression of estrogen’s functional units can disrupt many essential activities that rely on estrogen’s interaction with its nuclear receptors, leading to clinical consequences. Therefore, it is crucial to use appropriate treatments. The administration of DDZ increased the expression of both ER genes in the hippocampus, but it was not as effective as E2 in increasing ERα transcripts. It has been repeatedly reported that phytoestrogen exhibits a low affinity for binding to ERα, suggesting their weak estrogenic activities (10^−2^–10^−3^-fold) in comparison with E2 (36). Perhaps the cause of the lower effect of daidzein on the expression of estrogen receptor alpha can be attributed to this issue. However, there are also opposite results. For example, Jefferson *et al*. have reported that neonatal injection of genistein in the ovaries of mice did not change the ERβ expression (37). Another study also found that prenatal and neonatal dietary phytoestrogen decreased the ERα and ERβ mRNA expression in the mice ovaries (38). These reports could result from different administration methods, target tissues, or treatment periods.

Our stereological analysis of the OVX hippocampus revealed a significantly disrupted arrangement and degenerated shrunken pyramidal and granular cells in CA1 and DG, respectively. Treatment with DDZ caused a reduction in the population of shrunk pyknotic pyramidal cells, a dramatic increase in the total number of pyramidal cells, and induction of CA1 total volume in the OVX+DDZ hippocampus rats. However, the impact of DDZ on the volume and the number of granular cells in the DG region was not statistically meaningful when compared to either the OVX group or E2. Several studies have confirmed the positive effect of phytoestrogens treatment on the CA1 neuronal health. For instance, MacLusky *et al*. reported that phytoestrogens can reverse mitochondrial dysfunction in the hippocampal CA1 region and increase spine synapse density in this area (39). Another study also noted that maintenance of ovariectomized rats on chow containing high phytoestrogen levels for 9 weeks was associated with increased dendritic spine density in CA1 and prefrontal cortex pyramidal neurons (40). Our findings align with these previous studies. However, contrary to our results, Abd Ellatif has reported that the treatment with phytoestrogen panax ginseng improved the severity of histological alterations in the DG region (41). The CA1, CA2, CA3, and DG of the hippocampus contain various neurons and, thus, have different vulnerabilities and response times to the damages or hormones (42). Besides, it has indicated that the ERα and ERβ distribution in the subregions of the brain, including the hippocampus, varies (43). ERα has been more localized in the CA1 and CA3 pyramidal cell layer and the hilus of the DG (44). However, ERβ protein expression is mainly found in the CA2 and CA3 pyramidal layer, and a weak localization was reported in the DG granule cell layer. Therefore, it could be proposed that the higher affinity of DDZ to the ER β and the lowest presence of this receptor in DG granular cells led to a failure of DDZ to have an acceptable effect on this area during the treatment period of the OVX+DDZ rats.

Yang *et al*. declared that estrogen has a neuroprotective role as it enhances synaptogenesis by increasing the expression of BDNF, which is essential for synaptic plasticity and memory (45). It reported that the changes in BDNF levels significantly affect the neuronal morphology, basal neurogenesis, and volume of the hippocampal subfields (46). Tolwani *et al*. reported that an elevated expression of BDNF induced the dendrite complexity and morphological changes of DG and CA1 regions in the mice, probably due to Trk B receptor activation (47). The data obtained from this study showed that the suppressed expression of BDNF in OVX significantly increased after treatment with DDZ in the OVX+DDZ group. This finding aligns with a 2012 research that found that DDZ improved the hippocampus neuronal cell viability and proliferation mediated by the BDNF-Trk B pathway (48). It is also reported that the high dose of dietary supplement soy phytoestrogen treatment significantly increased BDNF concentration and the mRNA levels for BDNF and its Trk B receptors as well as the synaptic formation proteins, synaptophysin, spinophilin, and synapsin 1 in the hippocampal tissue of the ovariectomized rats (49).

By binding to the Trk B receptor, BDNF activates several intracellular pathways, including the phosphoinositide 3-kinase (PI3 K)/Akt/Bcl-2 pathway promoting newborn neuron survival and differentiation (50). Activated Akt could regulate several axial proteins, such as GSK-3β. GSK-3β protein targets various signaling cascades, impeding neuronal development and neurogenesis. It also augments the production of proinflammatory cytokines such as TNF-α and IL-6 in the BV-2 microglia cell line and human monocytes (51). Consistent with previous reports, our results indicated that ovariectomized rats exhibited higher levels of two inflammatory markers and increased GSK-3β protein levels in the hippocampus. The activity of GSK-3β is negatively regulated by the phosphorylation of Serine 9 residue that seems to depend on an estrogen-mediated activation of Akt. Thus, it could protect against neuroinflammation and apoptosis (52). Our western blotting analysis showed a remarkable increase in p-GSK-3β and the p-GSK-3β/GSK-3β ratio in the OVX+DDZ rats. DDZ and E2 significantly suppressed the expression of hippocampal TNF-α and IL-6 genes in the OVX+DDZ and OVX+E2 groups, respectively. Thus, it could be proposed that DDZ could inhibit ED-induced inflammation by inducing the phosphorylation of GSK-3β serin residues triggered by ER activation. However, for these hypotheses, more related molecules and genes, including PI3K and Akt genes, should be investigated, which, unfortunately, was beyond the scope of this study. 

The Bax /Bcl-2 ratio is considered an important apoptosis index. Previous research has demonstrated that peripheral inflammation could disrupt the Bax and Bcl-2 ratio (53) And that a decrease in the Bcl-2 protein promotes the opening of the mitochondrial permeability transition pore (54). The results of the present study showed that DDZ and E2 significantly up-regulated the expression of Bcl-2 and suppressed the Bax mRNA expression in the OVX rats, which accords with Mao *et al*.’s study (55). Additionally, as shown in Table 3 there was a significant suppression in the Bax/Bcl-2 ratio in the OVX+DDZ group compared with the OVX+E2 rats. Since DDZ had a more significant effect on the expression of the Erβ gene, the significance of this receptor as an anti-apoptotic factor in the hippocampus has been suggested. The key involvement of ERβ in the anti-apoptotic actions of DDZ in Parkinson’s disease induced by 6-hydroxydopamine has been previously demonstrated (56). Additionally, a novel regulatory site has been recently discovered in the promoter region of Tnfaip1 (tumor necrosis factor-induced protein 1) that binds to ERβ. This discovery suggests that estrogen or other selective ligands could be targeted to protect against brain inflammation and subsequent apoptosis (57). These findings confirm the data obtained from this study.

A review of previous studies and data obtained from the present study suggests three possible mechanisms through which DDZ may regulate apoptosis: direct interference with Bcl-2-dependent apoptotic processes and a decrease in the Bax/Bcl-2 ratio (58), suppressing the extrinsic death receptor-mediated apoptotic pathway (59), and inhibition of GSK-3β-mediated neuronal cell death (60). 

**Table 1:** Primer sequences used in real-time PCR. The table lists the forward (F) and reverse (R) primers for all target genes and one housekeeping gene utilized for real-time PCR

Gene of interest	Primer sequences (5'-3')
ERα	F: 5′-CCAAAGCCTCGGGAATGG-3′ R: 5′-AGCTGCGGGCGATTGAG-3′
ERβ	F: 5′-AGCTGCCAGGCCTGCCGAC-3′ R: 5′-AGCTGCACGGCCTGCCGAC-3′
BDNF	F: 5′-GTGACARTATTAGCGAGTGGG-3′ R: 5′-GGGTAGTTCGGCATTGC-3′
Bcl-2	F: 5′-CGACCTCTGTTTGATTTCTCCTG-3′ R: 5′-CTTTTCATATTTGTTTGGGGC-3′
Bax	F: 5′- TGCTACAGGGTTTCATCCAG-3′ R: 5′-TTGTTGTCCAGTTCATCGCC-3′
TNF-α	F:5´-GACCCTCACACTCAGATCATCTTC-3´ R:5'- TGCTACGACGTGGGCTACG -3
IL-6	F: 5′-CGAAAGTCAACTCCATCTGCC-3′ R:5′-GGCAACTGGCTGGAAGTCTCT-3′
GAPDH	F: 5′-GTCAGTGCCGGCCTCGTCTCATA-3′ R: 5′-GACCCTTTTGGCACCACCCTTCA-3′

A photomicrograph of Giemsa-stained sections (×400) has been used to study the CA1 and dentate gyrus (DG) subregion. The necrosis of the pyramidal neurons of CA1 and DG granular neurons in the OVX rats is illustrated by arrows. A disturbed arrangement with many degenerate-shrunken pyramidal cells and granular cells in CA1 and dentate gyrus is shown by black arrows. Sham and DDZ groups depicted a normal stereological manifestation

Sham: (Sham+Vehicle); DDZ: Daidzein+Vehicle; OVX: Ovariectomy+Vehicle; OVX+DDZ: Ovariectomy+daidzein; OVX+E2: Ovariectomy+17βestradiol; Vehicle: 90% corn oil and 10% ethanol

**Table 2:** Serum levels of the 17β-estradiol hormone in non-ovariectomized and ovariectomized groups prior to treatment

	Sham	DDZ	OVX	OVX+DDZ	OVX+E2
Serum E2 levels (g/ml)	72.1 ± 2	69.5 ± 1.1	10.3 ± 3**	11.1 ± 0.9**	9.53 ± 2.1**

Values are presented as mean±SEM

Sham: (Sham+Vehicle); DDZ: Daidzein+Vehicle; OVX: Ovariectomy+Vehicle; OVX+DDZ: Ovariectomy+Daidzein; OVX+E2: Ovariectomy+17βestradiol; Vehicle: 90% corn oil and 10% ethanol; SEM: standard error of the mean

**Significant difference with Sham group (*P<*0.01)

2A represents the expression changes of ERα, and 2B is related to Erβ mRNA expression. Values are presented as mean±SEM. ***(*P<*0.001), **(*P<*0.01), and * (*P<*0.05) vs corresponding Sham, ^†††^ (*P<*0.001), ^††^ (*P<*0.01) and ^†^(*P<*0.05) show the remarkable difference compared to the OVX rats, and $ (*P<*0.05) has been used to show the significant differences between OVX+DZD and OVX +E2. Vehicle (90% corn oil and 10% ethanol)

Sham: Sham+Vehicle; DDZ: Daidzein+Vehicle; OVX: Ovariectomy+Vehicle; OVX+DDZ: Ovariectomy+ daidzein; OVX+E2: Ovariectomy+17β-estradiol; SEM: standard error of the mean

The results are presented as mean±SEM. Expression levels of all transcripts were normalized with GAPDH and analyzed using the ∆∆CT method. ***(*P<*0.001) and *(*P<*0.05) vs corresponding Sham, ^††^ (*P<*0.01) and ^†^(*P<*0.05) show the remarkable difference compared to the OVX rats, and ^$^ (*P<*0.05) has been used to show the significant differences between OVX+DZD and OVX+E2. Vehicle (90% corn oil and 10% ethanol)

Sham: Sham+Vehicle; DDZ: Daidzein+Vehicle; OVX: Ovariectomy+Vehicle; OVX+DDZ: Ovariectomy+daidzein; OVX+E2: Ovariectomy+17β-estradiol; SEM: standard error of the mean

4A and 4B represent the effects of DDZ on the expression levels of two hippocampal inflammatory cytokines genes, TNFα (4. A) and IL-6 (4. B), respectively. The results are presented as mean±SEM. ***(*P<*0.001), **(*P<*0.01), and *(*P<*0.05) vs corresponding Sham, ^††^ *P<*0.01 shows the remarkable difference compared to the OVX rats, and ^$^ (*P<*0.05) has been used to show the significant differences between OVX+DZD and OVX+E2. Vehicle (90% corn oil and 10% ethanol)

Sham: Sham+Vehicle; DDZ: Daidzein+Vehicle; OVX: Ovariectomy+Vehicle; OVX+DDZ: Ovariectomy+daidzein; OVX+E2: Ovariectomy+17β-estradiol; SEM: standard error of the mean

Expression levels of the Bcl-2 and Bax genes after treatment with daidzein are presented in Figures 5.A and 5.B, respectively. The results are presented as mean±SEM. ***(*P<*0.001), **(*P<*0.01), and *(*P<*0.05) vs corresponding Sham, ^††^ (*P<*0.01) and ^†^ (*P<*0.05) show a remarkable difference compared to the OVX rats, and ^$^ (v0.05) has been used to show the significant differences between OVX+DZD and OVX+E2. Vehicle (90% corn oil and 10% ethanol)

Sham: Sham+Vehicle; DDZ: Daidzein+Vehicle; OVX: Ovariectomy+Vehicle; OVX+DDZ: Ovariectomy+ daidzein; OVX+E2: Ovariectomy+17β-estradiol; SEM: Standard error of the mean

**Table 3:** The ratio of Bax/Bcl-2 mRNA in the hippocampus

**Groups**	**Bax/Bcl-2 ratio in the hippocampus**
**Sham**	1.01 ± 0.01247
**DDZ**	1.12 ± 0.01112
**OVX**	12.7 ± 0.02178 **
**OVX+DDZ**	2.06 ± 0.01541 †$
**OVX+E2**	5.76 ± 0.01435*†

Values are presented as mean±SEM. **Significant difference with Sham group (*P<*0.01), *(*P<*0.05). and ^†^ (*P<*0.05) show significant differences compared to the sham and OVX groups respectively. $(*P<*0.05) shows the significant difference between OVX+DDZ and OVX+E2 rats. Vehicle (90% corn oil and 10% ethanol)

Sham: Sham+Vehicle; DDZ: Daidzein+Vehicle; OVX: Ovariectomy+Vehicle; OVX+DDZ: Ovariectomy+ daidzein; OVX+E2: Ovariectomy+17β-estradiol; SEM: Standard error of the mean

6 A shows the representative blot imaging for the hippocampal GSK-3β protein and P-GSK3β. The protein level of GSK-3β and p-GSK-3β normalized to the tubulin protein, and the P-GSK-3β/ GSK-3β ratio is depicted in Figures 6.B, 6.C, and 6 D, respectively. The results are presented as mean±SEM. **(*P<*0.01) and *(*P<*0.05) vs corresponding Sham, ^††^(*P<*0.01) and ^†^(*P<*0.05) show the remarkable difference compared to the OVX rats, and ^$ ^(*P<*0.05) has been used to show the significant differences between OVX+DZD and OVX+E2. Vehicle (90% corn oil and 10% ethanol)

Sham: Sham+Vehicle; DDZ: Daidzein+Vehicle; OVX: Ovariectomy+Vehicle; OVX+DDZ: Ovariectomy+ daidzein; OVX+E2: Ovariectomy+17β-estradiol; SEM: standard error of the mean

7A depicts the changes in the volume of the CA1 region, 7B indicates the changes in the volume of DG, 7C shows the total number of pyramidal cells in the CA1 region, and Figure 7. D illustrates the total number of DG granular cells. The results are presented as mean±SEM. ***(*P<*0.001) and * (*P<*0.05) vs corresponding Sham, ^†††^ (*P<* 0.001) and ^†^ (*P<*0.05) show the remarkable difference compared to the OVX rats, and ^$^ (*P<*0.05) has been used to show the significant differences between OVX+DZD and OVX+E2. Vehicle (90% corn oil and 10% ethanol)

Sham: Sham+Vehicle; DDZ: Daidzein+Vehicle; OVX: Ovariectomy+Vehicle; OVX+DDZ: Ovariectomy+ daidzein; OVX+E2: Ovariectomy+17β-estradiol; SEM: standard error of the mean

## Conclusion

Our data suggests that by enhancing the estrogen receptor gene expression, especially ERβ, and involving two important molecules, BDNF and GSK-3β, the phytoestrogen daidzein could reduce the neuroinflammation, neuro-apoptosis, and stereological changes in the CA1 subregion induced by estrogen deficiency in the hippocampus. Although daidzein was not as effective as estradiol in reversing ovariectomy-induced stereological changes in the DG subregion, changing the dosage or duration of treatment might make it a suitable alternative to HRT.

## Data Availability

Data used in this study are available upon request.

## Declaration

All the authors declare that the present manuscript has not used AI tools or technologies to prepare this manuscript.
